# DNA and RNA analyses in detection of genetic predisposition to cancer

**DOI:** 10.1186/1897-4287-10-17

**Published:** 2012-12-04

**Authors:** Grzegorz Kurzawski, Dagmara Dymerska, Pablo Serrano-Fernández, Joanna Trubicka, Bartłomiej Masojć, Anna Jakubowska, Rodney J Scott

**Affiliations:** 1 International Hereditary Cancer Center, Department of Genetics and Pathology, Pomeranian Medical University, Szczecin, Poland; 2 Department of Medical Genetics, the Children’s Memorial Health Institute, Warsaw, Poland; 3 The Discipline of Medical Genetics, Faculty of Health, University of Newcastle and the Hunter Medical Research Institute, Newcastle, Australia; 4 The Division of Genetics, Hunter Area Pathology Service, John Hunter Hospital, Newcastle, Australia

**Keywords:** Constitutional changes, Hereditary cancer, Techniques, Diagnoses

## Abstract

During the past decade many new molecular methods for DNA and RNA analysis have emerged. The most popular thus far have been SSCP, HET, CMC, DGGE, RFLP or ASA, which have now been replaced by methods that are more cost effective and less time consuming. Real-time amplification techniques and particularly those with the capacity of multiplexing have become commonly used in laboratory practice. Novel screening methods enable the very rapid examination of large patients series. Use of liquid handling robotics applied to the isolation of DNA or RNA, the normalisation of sample concentration, and standardization of target amplification by PCR have also contributed to a reduced risk of sample contamination and have resulted in laboratory analysis being easier and faster.

The aim of this study is the introduction of a few modern techniques, most commonly used in detection of genetic predisposition to cancer.

## 

Several genes have been identified which, if mutated, are associated with increased predisposition to cancer [1]. Persons harbouring mutations in these genes have an altered risk of cancer from a few percent to as much as 90% compared to the non-affected controls. A series of genes associated with an inherited predisposition to cancer and those most frequently examined in clinical practice are summarised in Table 1.

**Table 1:** Genes associated with predisposition to cancer family syndromes. The table contains genes studied the most frequently in our centre

**GENE LOCALISATION**	**PREDISPOSITION TO MALIGNANCIES**	PENETRANCE*
*Rb1*[2] 13q14	retinoblastoma	up to 90%
*BRCA1*[3] 17q21	breast cancer	up to 80%
*BRCA2*[3] 13q13	ovarian cancer	
	prostate cancer	
	colon cancer	
*VHL*[4] 3p25	haemangioblastoma of the	up to 80%
	cerebellum and retina	
	kidney cancer	
	pheochromocytoma	
*MSH2*[5] 2p21	colon cancer	up to 90%
*MLH1*[5] 3p22	endometrial cancer	for male
*MSH6*[5] 2p16	cancer of the stomach,	up to 70%
	cancer of the biliary tract	for female [6]
	small bowel cancer	
	ovarian cancer	

*probability of malignancy during lifetime among mutation carriers.

Several molecular methods have been developed that are specifically designed for detecting mutations that can be further subdivided into methods aimed at detecting:

● new mutations

● known mutations

## Detection of new mutations

The diagnosis of mutations in appropriately selected cases using pedigree and clinical data is justified in clinical practice, even though mutation detection techniques can be complex, time-consuming and expensive.

### Requirements of DNA mutation analyses

The principal requirements for mutation analysis include:

● DNA isolation,

● amplification of gene fragments, usually comprising only coding sequences (but this is beginning to encompass the promoter regions of genes)

● preliminary detection of changes within amplification products using screening techniques

● Southern analysis and multiplex ligation-dependent probe amplification

● high resolution melting analysis

● sequencing and pyrosequencing

### DNA isolation

Constitutional DNA is usually isolated from whole blood and less frequently from other tissues. Analysis of constitutional DNA allows the detection of mutations that occur in all nucleated cells of patients. Optimal DNA isolation is especially effective if the sample for analysis is collected within 48 hours of manipulation. Good results, however, can be achieved even after a few days of blood storage at room temperature or even as much as a few years if the sample is kept at temperatures below zero. If fresh tissue is not available, DNA isolation can be performed from formalin fixed paraffin embedded tissue blocks, although attaining unequivocal results using such material is more difficult and sometimes even impossible. DNA isolation requires elimination of proteins from cellular lysates. Using the phenol-chloroform method this is achieved by digestion with proteinase K and extraction in a mixture of phenol and chloroform. Finally, nucleic acids are extracted using ethyl- or isopropyl- alcohols. This technique is used, however, only occasionally even though it produces exceptionally clean and non-degraded DNA (in practice it is now used mainly for DNA isolation from paraffin blocks). Other techniques have been developed that are less laborious and easier to automatate. They are primarily based on binding DNA to a synthetic bead that can be easily separated from other cellular components (e.g. with dyne beads) and washed in order to separate out clean and amplifiable DNA.

### Amplification of gene fragments

Targeted DNA fragments are amplified using the polymerase chain reaction. The reaction mixture includes: DNA template (usually genomic DNA), DNA polymerase, a pair of specific primers for the gene segment to be analysed, deoxyribonucleotide triphosphates and a reaction buffer. This mixture is exposed to cyclic changes of temperature which activate a heat sensitive DNA polymerase that generates a new complementary strand of DNA from a single strand template. Each cycle includes: denaturation, annealing initiation and DNA synthesis. After 22 cycles, assuming 100% efficiency, the copy number of the amplified fragment is increased one million-fold.

### Preliminary detection of changes within amplification products using screening techniques

Most screening techniques take advantage of the unique nature of double stranded DNA. At temperature sufficient to “melt” DNA from a double strand to a single strand re-annealing can occur. In the presence of a mismatch the annealing temperature decreases and the double strand molecule falls apart much more easily than when there is a perfect match. DNA-SSCP (single strand conformational polymorphism) was one of the first methods to take advantage of differences in annealing temperature and was the most popular technique for the detection of differences in amplification products [7]. Other techniques using this principle include HET (heteroduplex analyses) [8], CMC (chemical mismatch cleavage) [9], DHPLC (denaturing high-performance liquid chromatography) [10] and DGGE (denaturing gradient gel electrophoresis) [11].

### DHPLC (denaturing high-performance liquid chromatography)

**Figure 1:**
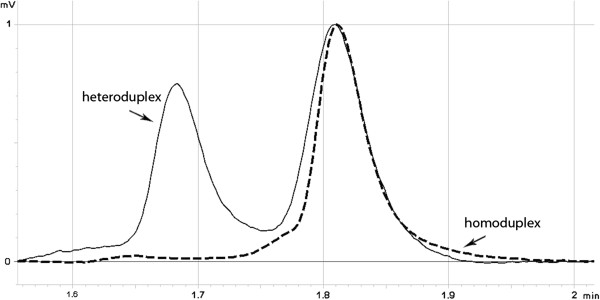
**DHPLC elution profile characteristic for c.1786_1788delAAT mutation in *MSH2 (mutS homolog 2) *gene (solid line) compared to ‘wild’ type (dashed line).**

At present, the best and the most frequently applied DNA screening technique for the initial detection of changes is DHPLC [10,12-15]. This is based on the same principals as HET but includes high-resolution DNA specific chromatography columns that under appropriate conditions can separate DNA sequences differing by as little as one base. The melting conditions are a function of the sequence that is being interrogated and they can take some time to optimize. Amplified DNA fragments are separated in a gradient of denaturing agent. (The key to DHPLC is the solid phase, which has differential affinity for single and double-stranded DNA). Under sub-denaturing conditions, heteroduplexes have a lower affinity than homoduplexes to the solid phase of the column and it is easier to elute them. Separation is monitored by UV absorption measured at 260 nm. The elution profile (Figure 1) is characteristic and reproducible for a given change and allows differentiation between new changes and known mutations or polymorphisms.

Based on reported data [16] and our own experiences [17] we can state that DHPLC combines the advantages of several methods. Its sensitivity approaches 100% [10,14,15]. At the same time the cost is relatively low (reagent costs per sample ~ 5–10 Euro). The method is rapid, and if an auto-sampler is used, it can allow up to 200 samples per day to be analysed.

### DNA sequencing

Sequencing is considered to be the most sensitive technique for the detection of changes in genomic material, allowing at the same time their full characterisation and it is often considered to be the “Gold Standard” of mutation detection.

Since the 1990’s, significant progress in DNA sequencing technologies has been achieved by the introduction of automated DNA fragment analysers, for which the identification of particular nucleotides is based on base specific fluorescent dyes that are activated by laser emission. Each nucleotide (A, C, G, T) is labelled with a different fluorescent dye, which is detected by a laser targeting the excitation wavelength of the fluorescent dye and the resultant emission identified by a photomultiplier.

The most convenient sequencing assay relies on a cyclic sequencing [18] based on the classical Sanger method. During the analysis the PCR amplified sequences of the target products of both the forward and reverse DNA strands are assessed. Any legitimate change is detected in both DNA strands. The sequencing procedure comprises several stages that include:

● preparative PCR – amplification of the target fragment of the gene using specific primers pairs,

● asymmetric PCR – separate amplification with each of the primers using a mixture of amplification primers and fluorescent dye-labelled dideoxynucleotides (once incorporated the sequence reaction stops),

● fragment size separation by electrophoresis in denaturing polyacrylamide gel with simultaneous detection and registration of products,

● Data analysis of the results using computer programs.

**Figure 2:**
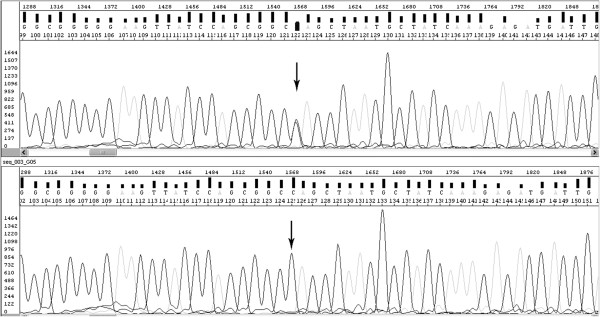
**Chromatograms for DNA sequencing: studied sequence with c.83C>T mutation in *MLH1 (mutL homolog 1) *gene (upper) and ‘wild’ sequence (below).**

During asymmetric PCR all possible oligonucleotides of different lengths, complementary to the template and containing fluorochromes at the 3’-end are created. They are size separated by electrophoresis and the base order of coloured nucleotides can be read as the sequence complementary to the template. The detected DNA sequence is compared with the wild type sequence (Figure 2) available in databases such as GenBank and EMBL, and the type of change can be precisely described.

Currently, the major DNA sequencing companies provide modern automated DNA sequencing instruments (DNA sequencers) that can simultaneously sequence up to 96 samples using capillary electrophoresis. Recent progress in this discipline (Sanger-like sequencing) is based not only on the increased number of simultaneously analysed samples but also on an improved “chemistry” that includes a higher resolution capillary gel composition that has resulted in the ability to accurately sequence almost one thousand bases of amplified DNA.

More recently commercially available next-generation sequencing (NGS) platforms have appeared that promise to significantly reduce DNA sequencing costs. Many different types of instrument have been produced that are still being perfected for diagnostic applications, such as the Roche 454 and GS Junior system (http://www.454.com) and the Illumina Genome Analyzers, HiSeq and MiSeq (http://www.illumina.com), Ion Proton and Ion torrent (http://www.iontorrent.com). Although these are designed mainly for sequencing whole genomes, some of them have applications similar to traditional sequencing such as targeted re-sequencing and mutation detection [19]. The advantage of this new approach to DNA sequencing is that selected panels of genes can be screened for germline or somatic mutations in a single reaction [20-22]. This is especially important for cancer predispositions, as it will be possible, for example, to screen all genes associated with breast cancer risk in a single reaction. An even greater potential which would herald a total revolution in molecular diagnostics are the new techniques aimed at reading a sequence of native DNA without the need for pre-amplification, termed True Single Molecule Sequencing (http://www.helicosbio.com), SMRT TECHNOLOGY (http://www.pacificbiosciences.com) or the GridION system based on NanoPore technology announced by Oxford NanoPore Technologies, based in Oxford, UK.

The 454 Genome Sequencer 20 (454 Life Sciences, Roche Applied Sciences, Indianapolis, IN) was the first commercially available, next-generation sequencing instrument, with the current 454 FLX+ system being the recently upgraded version. These new sequencers, FLX+ machines, rely on real time sequencing by simultaneous synthesis of many DNA fragments (around 700 hundred base pairs in length). They apply pyrosequencing technology that detects base additions by luminescence as a result of ATP degradation during the sequencing reaction and can read up to 900 Mb at a time.

**Figure 3:**
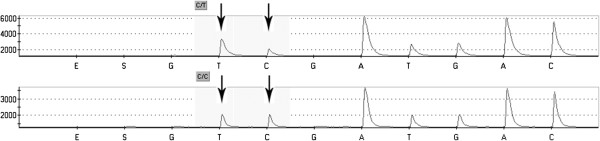
**Pyrograms: patient with c.2932C>T in *APC (adematous polyposis coli) *gene (upper) and ‘wild’ type (below).**

Pyrosequencing [23,24] uses a single strand of a DNA fragment as the template on which synthesis of a complementary strand is performed through the addition of 4 different deoxynucleotide triphosphates (dNTPs). The addition of each base is associated with the liberation of pyrophosphate which is transformed into ATP using sulfurylase and adenosine-5’-phosphosulphate. ATP is used by luciferase for the transformation of luciferin into oxyluciferin. During this reaction light is generated with an intensity proportional to the amount of pyrophosphate produced. The emitted light is registered by a CCD camera and transformed into peaks on a pyrogram (Figure 3). The same reaction scheme is used for each dNTPs. If the added nucleotide is not complementary to the template it is not included in the newly synthesised strand and pyrophosphate is not created. The presence of a light signal is the requisite for adding a new nucleotide to a given sequence. Based on our own experience the major disadvantage of pyrosequencing technique is the difficulty in determining the number of incorporated nucleotides in homopolymer regions. In homopolymer regions of more than 5 nucleotides, the estimation of complementary added nucleotides is not possible due to a nonlinear light response. However the problem can be solved by using nucleotide reversible terminators (NRTs), analogues modified by attaching a cleavable fluorophore to the base and a chemically reversible moiety to the 3’ cap (3’-*O*-allyl or 3’-*O*-(2-nitrobenzyl)). During the extension step when the complementary NRT is incorporated, the reaction is temporarily terminated and resumed only when the capping moiety is removed (by de-allyation or laser irradiation). In this way, when analysing the results, each peak correspond to each incorporated nucleotide and homopolymeric regions can be clearly identified [25].

Despite the appearance of an application to the sequencing of genes related to hereditary cancer syndromes [26,27] and significantly lower cost [28] the use of this technology has not yet become widespread. There are also inherent problems in NGS approaches as overall the instrumentation is inaccurate and as such accuracy is only achieved by repeating reactions many times such that fold coverage must be high before statistical confidence in the results can be achieved.

### Southern method and MLPA (multiplex ligation-dependent probe amplification)

A technique very popular in the past for the detection of large rearrangements was Southern blotting, described for the first time by E. M. Southern in 1975. Currently this method has been almost completely replaced by MLPA (multiplex ligation-dependent probe amplification) [29] for the detection of large genomic rearrangements targeting specific cancer predisposition genes. MLPA is based on the ligation of specific probes and their subsequent amplification that allows an assessment of exon copy number to be made either fore a single exon or an entire gene. On this basis, conclusions can be drawn concerning deletions or duplications of gene fragments or whole genes.

**Figure 4:**
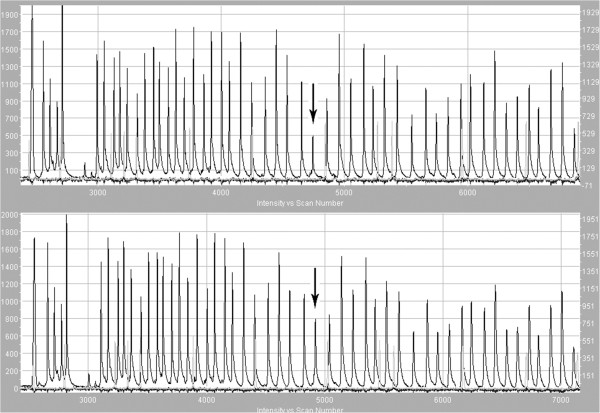
**Result of MLPA electrophoresis of a sample with deletion of exon 9 in *MSH2 (mutS homolog 2) *gene (upper) and ‘wild’ type (below).**

In this technique many probes are used simultaneously in a single reaction. Probes matching the sequences complementary to exon sequences also contain primer sequences and one of each of the pairs additionally contains a unique insertion sequence called a stuffer sequence. Hybridising sequences of each pair of probes match neighbouring DNA fragments occurs and only if this is complete can ligation take place. After probe hybridisation to the template, the DNA fragments are ligated and denatured. The dissociated ligated probe containing primer sequences is then amplified using PCR. The presence of stuffers of different lengths allows differentiation of the products that have labelled different targets (in this case, exons with the gene in question), and the amount of product is proportional to the copy number in the template. Each peak corresponds to the product of amplification of specific ligated pairs of probes (Figure 4). Relative differences in the height or area of the peak indicate quantitative (sometimes qualitative) changes of a target sequence for the probe. In the event a single exon appears to be missing in the gene in question, caution must be made as it may be due to the presence of a polymorphism under the target sequence. To overcome this, all single exon deletions should be sequenced to confirm that the primer binding sites are wild type and do not harbour a polymorphism that would disrupt the assay integrity.

The advantages of this technique are that only a small amount of DNA is necessary to perform analyses and that efficiently reproducible results may be achieved even from degraded genetic material.

Commercially available probes include those for the most important genes associated with a high risk of tumours, such as: *ATM, BRCA1, BRCA2, CHEK1, MLH1, MSH2, MSH6, PMS2, EPCAM, APC, FANCA, FANCD2, PTCH, BMPR1A, SMAD4, TP53, CDH1, MEN1, NF1, NF2, STK11, SMARCB1, RB1, CDKN2A-CDKN2B, WT1.*

### HRMA (high resolution melting analysis)

This real-time PCR based method can be used for detection of SNPs as well as for large rearrangements. All mutations (small and large) can be screened simultaneously in one assay, which reduces screening time. The basis of the genotyping is a unique pattern of melting curves.

**Figure 5:**
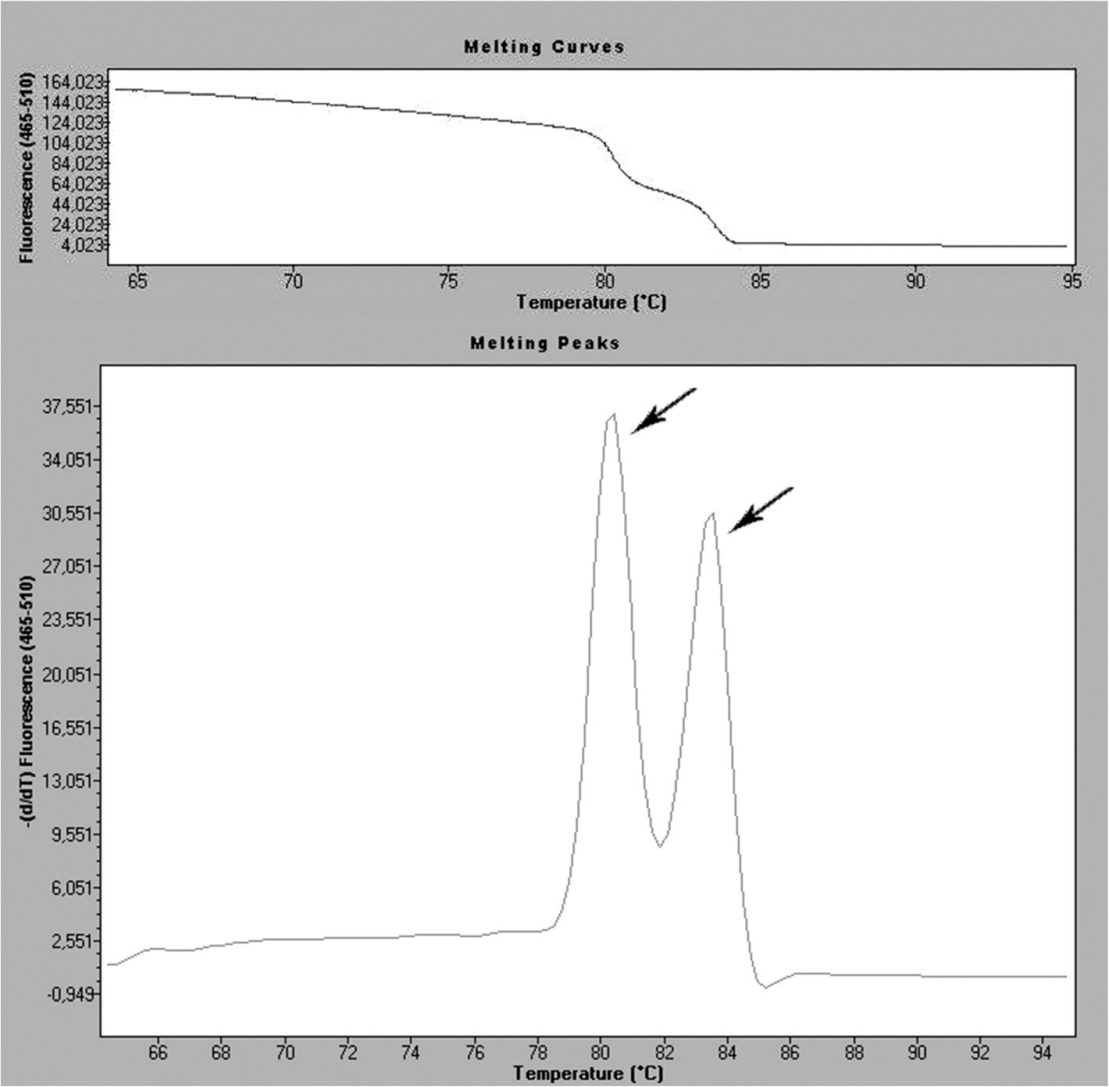
**Melting curve and melting peaks charts of a heterozygous mutation in exon 23 in *NF-1 (neurofibromatosis 1) *gene during screening by HRMA.**

The first step of the analysis is real-time PCR with fluorescent dye, mostly SYBR Green, LCGreen or Syto 9 [30]. That allows monitoring the amplification of the DNA template, since fluorescence intensity is proportional to the amount of double-strand DNA (dsDNA). After overheating, the melting behaviour of the PCR products is monitored by plotting the changes in fluorescence that occur by denaturating double-strand DNA (dsDNA) (Figure 5). The pattern of melting temperature (Tm) differences allows the discrimination of homo- and heterozygotes. The main problem of HRM is that differences in the shape of melting curve can easily identify heterozygotes, but may not distinguish all homozygotes [31]. However, the high sensitivity and specificity, low cost, small amount of DNA required (<5 ng) and the simplicity of the method are prominent features that make HRM a great candidate as a new screening method for mutation detection for genetic cancer predispositions [30,32].

### RNA analyses

The advantages of RNA analyses are mainly due to the possibility of detecting a mutation using a lower number of reactions (this is related to the shorter length of RNA in comparison to DNA). So far the main disadvantages of these techniques have included difficulties in achieving reproducible results, lower stability of RNA with mutations and difficulties in interpretation as a result of the occurrence of RNA alternative splicing.

RNA analyses includes three main stages:

● RNA isolation,

● amplification of coding parts of the genes,

● detection of changes in amplification products.

### RNA isolation

In the majority of laboratories, RNA is isolated from peripheral blood lymphocytes. RNA isolation is performed similarly to DNA isolation. However, due to the widespread presence of thermostable RNAses in tissues, RNA isolation has to be performed more carefully. It is very popular to use the isolation method by Chomczyński [33] from cellular lysates in a solution of guanidine thiocyanate (RNAse inhibitors) followed by extraction in a mixture of phenol and chloroform. The slightly acidic pH of phenol leads to removal of not only proteins but also of DNA, which under such conditions is practically insoluble.

### RT-PCR (reverse transcriptase PCR)

RNA can be reverse-transcribed into cDNA (complementary DNA) using reverse transcriptase and then amplified using standard PCR conditions. Since RNA does not include introns only a few pairs of primers are enough to amplify the entire coding region of the selected gene. Primers should be designed to overlap one another so that the entire gene can be analysied. Analysing cDNA on regular agarose gels usually allows detection of RNA abnormalities, which are the result of deletions or insertions of a few base pairs or splice site mutations.

### Detection of alterations in amplification products

Products of the RT-PCR reaction can be analysed using all of the techniques described above and by using the in vitro transcription translation (IVTT) assay, also known as the protein truncation test (PTT) [34,35]. RT-PCR is the first stage of PTT. For PTT one primer includes sequence information that is used for initiating transcription into cDNA as well as translation – to allow in vitro protein synthesis from a cDNA template. After translation the protein product is sized separated by electrophoresis and transferred onto a nylon membrane., The length of the synthesised protein is assessed against the theoretically expected size and any products that are smaller (or larger) that expected will be identified. This will include the effects of large deletions or insertions as well as single nucleotide mutations leading to stop coding (TGA, TAA or TAG) or splicing mutations. The disadvantage of PTT is its limitation in detecting missense mutations.

Finally, it is estimated that even if we use all known tests for the direct detection of mutations their sensitivity does not exceed ~ 70-80%, most lekiely due to a lack in the diagnosis of changes associated with the regulation of gene expression.

## Detection of known mutations

There is accumulating knowledge about the type and frequency of mutations predisposing to tumours, which can be population specific. These include both founder mutations and recurrent mutations in families of a given ethnic group. DNA tests aiming to detect all known founder mutations within a population are highly valuable due to an unusually high economical effectiveness. genes such as *BRCA1, MLH1, MSH2* and *VHL* have been studied intensively and it is known which mutations should be studied first prior to more expensive and extensive screens [36-38] in many populations.

The most frequently applied DNA tests used for detecting known mutations include the following techniques:

● restriction fragment-length polymorphism-(RFLP) PCR

● allele-specific amplification

● real-time PCR with TaqMan probes/SimpleProbes

● matrix assisted laser desorption/ionization time of flight

● SNaPshot genotyping

● strip assay based on primer extension reaction

### RFLP-PCR (restriction fragment-length polymorphism-PCR)

Restriction enzymes identifying specific sequences of the PCR products are described. This approach can be used for the detection of all mutations that lead to loss or creation of restriction sites. Amplified products containing a particular change are digested by restriction enzymes and then size separated on agarose or polyacrylamide gels.

### ASA (allele-specific amplification) – detection of mutations using specific oligonucleotides

A conventional variant of this technique uses not only flanking primers but also a primer fully complementary to the allele with a mutation or a primer that is complementary to the allele with a mutation and another to the wild allele, followed by agarose gel electrophoresis. Primers are localised in such a way that different PCR products are of different length depending on the genotype of the examined DNA sample. This technique, popular in the past, is now applied mainly in small laboratories without specialised equipment.

The modern version of this technique uses short allele-specific probes and real time PCR [39,40]. This allows very fast analysis of many DNA samples. Technology using a template with oligonucleotides immobilised on a solid phase can be considered as a modern version of ASA. A big advantage of this technology is automation and the possibility of analysing up to a few thousand known mutations. In many countries the use of such technology is limited due to high costs.

### Real-time PCR

One of the most modern and more frequently applied techniques in molecular biology is real time PCR which allows for the monitoring of the quantity of PCR products in each amplification cycle. A modification of this technique based on the application of fluorescent probes and complementary to the sequences of examined DNA fragments can be also applied to the identification of known genetic changes.

There are several systems based on this technique that differ in the type of probe used for detection of the targeted changes. Among them, systems applying TaqMan and Simple probes stand out.

### TaqMan probes

**Figure 6:**
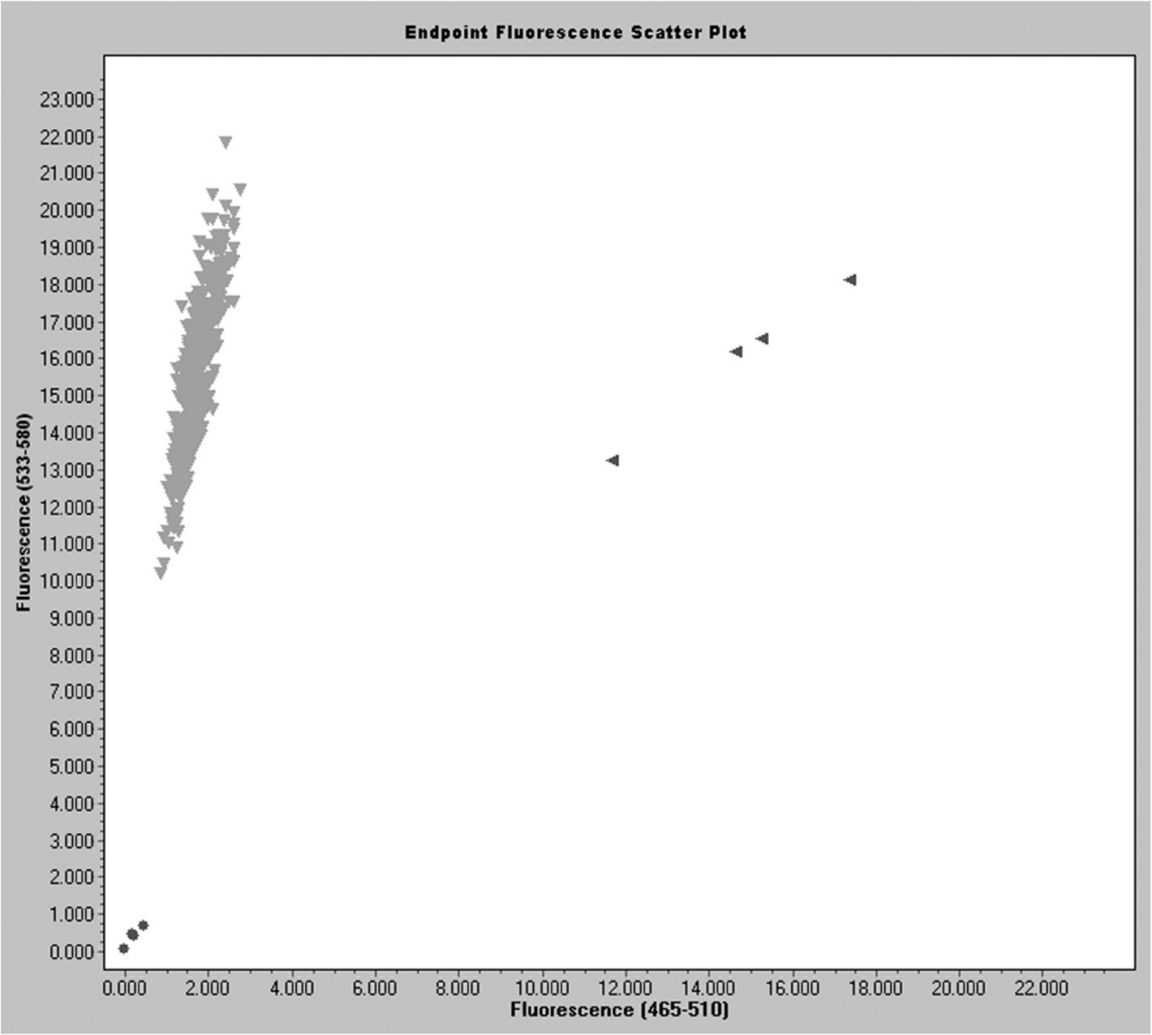
**TaqMan allelic discrimination for *MLH1 (mutL homolog 1) *c.677 G>T assay.** The G/G genotype is shown as light triangle-shaped dot, the G/T genotype as dark triangle-shaped dot, blank control as round-shaped dot.

Each of the two probes specific to the amplified fragment (to ‘normal’ and ‘mutant’ DNA variant) used in this system is labelled at the 5’ end by reporter dye: FAM (6-carboxy-fluorescein), VIC, HEX (hexachloro-6-carboxy-fluorescein), TET (tetrachloro-6-carboxyfluorescein) or JOE (2,7-dimethoxy-4,5-dichloro-6-6-carboxy-fluorescein) and at the 3’ end by quenching dye: TAMRA (6-carboxy-tetramethyl-rhodamine) or DABCYL (4-(4’-dimethylaminophenylazo)benzoic acid). The short distance between dyes within the same probe leads to quenching of the fluorescence. During the PCR reaction at the stage of primer annealing, a labelled probe is linked specifically to a specific template between sites of primer hybridisation. Its 3’ end is unavailable, which means that in the next stage – primer extension – this probe cannot be elongated with primers. Polymerase used in this system shows 5’-3’ activity and degrades the probe during DNA strand building. This leads to release of the reported dye from the quenching dye and causes increased fluorescence of one (in case of homozygote) or two (in case of heterozygote) reporter dyes (Figure 6). This process occurs during each cycle, causing an increase of the fluorescence signal from each cycle, which allows signal detection at each moment of the reaction.

Probes used in this system are 20–40 nucleotides in length. The number of GC pairs in their sequence is 40-60%. Probes should not include single nucleotide repeats, particularly guanine. Also the sequence of the probe should not be complementary to primer sequences or to sequences of the template at the sites of the annealing primers. It is important that the probe does not include guanine at the 5’ end, because its presence quenches reporter dye even after separation of it from quenching [41]. Modification of this system can be achieved by applying a TaqMan probe (described as a Minor Groove Binder or MGB type), in which the group MGB is fixed to the 3’ end. It protects stabilisation of probe annealing by matching the complex resulting from probe and template DNA. Interaction of the MGB group with the probe-template complex increases the temperature of probe melting by 15-30°C, which allows the use of probes of much shorter sequence (14–18 nucleotides). This is valuable during analyses of single nucleotide polymorphisms because it is easier to destabilise short probes under the influence of nucleotide changes in the examined sequence [42].

### Simple probes (guanine quenching probes)

**Figure 7:**
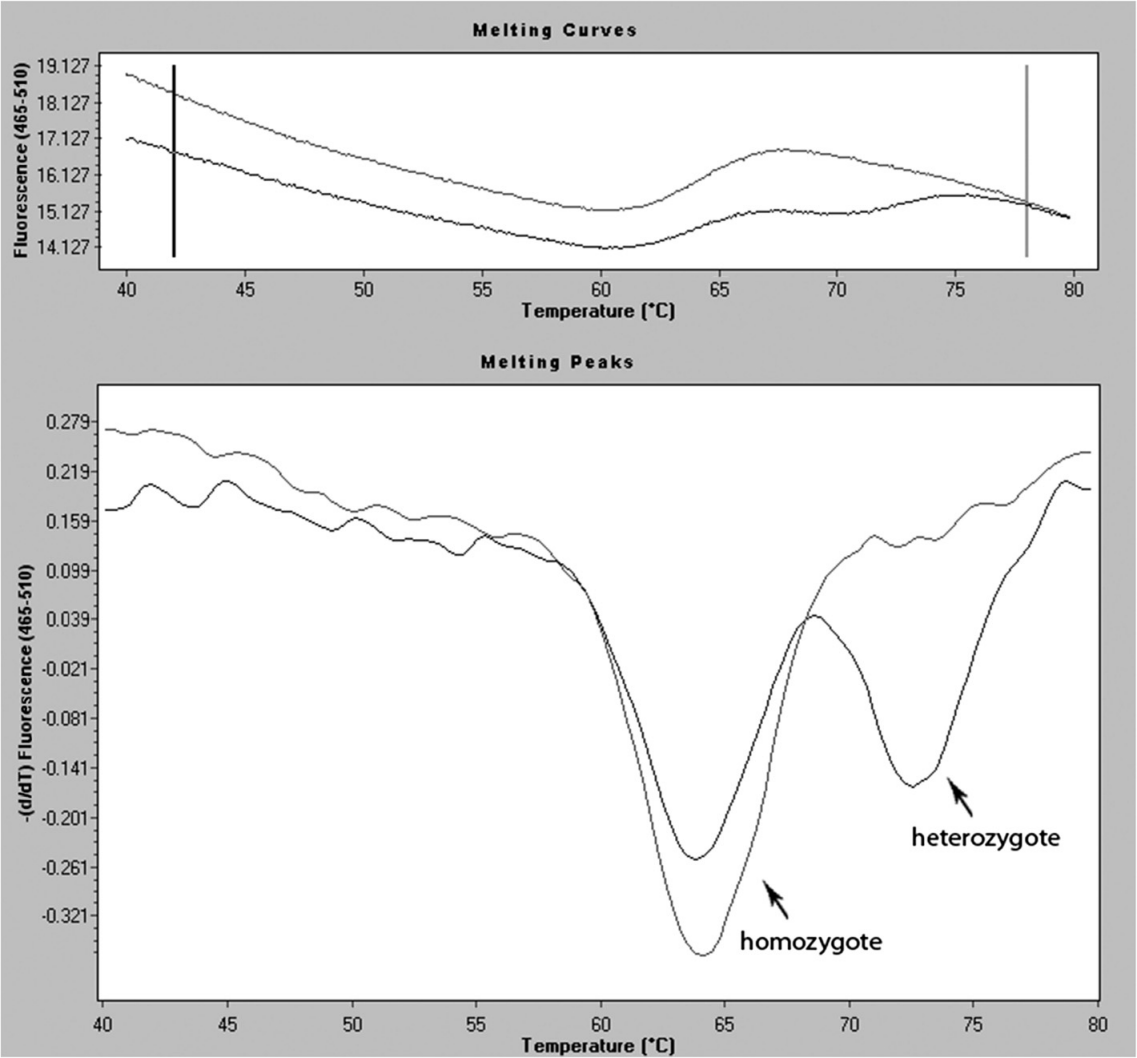
**Melting curve and melting peak charts for mutation in c.178 G>T in *MC1R *(*melanocortin 1 receptor) *gene (heterozygote) compared with wild type (homozygote) using real-time PCR with Simple probes.**

Guanine shows features of quenching fluorescence of such molecules as FAM or JOE. In this technique a short one-strand DNA fragment of 20–30 nucleotides in length (molecular probe) with a sequence complementary to the examined DNA containing the change/mutation labelled at 5’ or 3’ by fluorescent dye (FAM or JOE) is used. This technique allows identification of heterozygote and homozygote variants or mutations by measuring the increase in fluorescence achieved in the temperature gradient (Figure 7). The probe hybridising with the examined sequence is usually at a higher melting temperature if it hybridises with the fully complementary strand and lower melting temperature if an unpaired nucleotide is found in the probe. Reading the fluorescence levels during the temperature increase in the range 40-80°C allows the specific DNA change to be identified. Application of complementary fluorescent probes has several advantages. They include: high sensitivity, short time and full automation of the analyses and low risk of contamination by performing all stages in closed wells. The disadvantages of this technique include the necessity of protecting probes for particular sequences and low generality of experimental conditions [41].

### MALDI-TOF (matrix assisted laser desorption/ionization time of flight)

**Figure 8:**
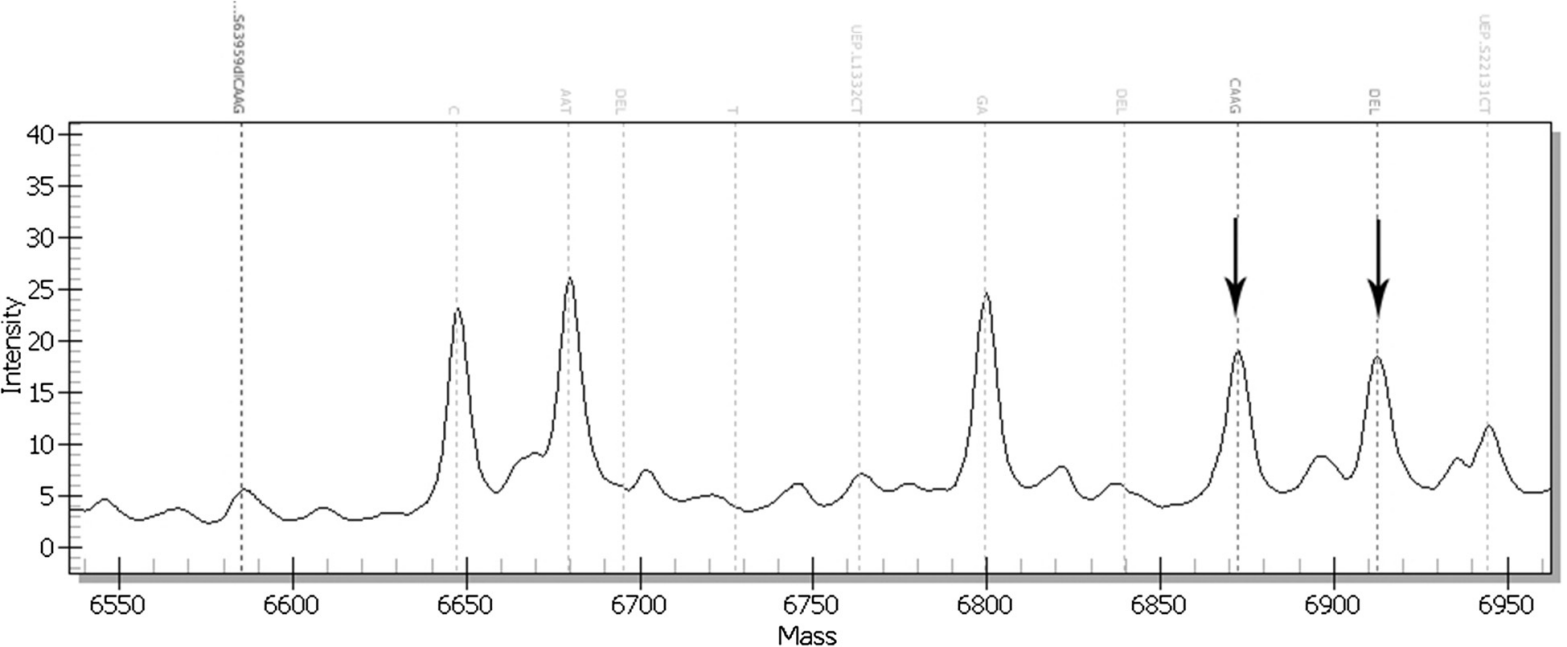
**Mass spectrum of a sample with c.3959_3962delCAAG mutation in *MSH6 (mutS homolog 6) *gene.** Each peak corresponds with each DNA variant.

MALDI-TOF is one of the techniques of mass spectrometry, applied for detection of changes within examined DNA fragments. Most frequently it is used for analyses of single nucleotide polymorphisms (SNPs). Analyses are preceded by stages based on PCR amplification. The first leads to amplification of selected fragments (in the multiplex version) containing the SNP to be examined. The second is asymmetric (one primer complementary to the sequence close to the polymorphic site), similarly to sequencing with application of dideoxy-nucleotides. After cleaning using ion exchange resin the samples are placed on Spectrochip and finally stimulated using a laser impulse for ion excitation. Total analysis is performed under vacuum, which means that the ion mobility is not disturbed by colliding gas particles. The speed of movement of the exited ions from the examined DNA sample is analysed by a detector that measures the time of ion flight. Ions from larger mass reach the detector more slowly than ions from a smaller mass. Nucleotide differences occurring in examined DNA samples are correlated with mass, which allows their differentiation (Figure 8). Separation of analysed particles is performed based on the ratio of ion mass to their electric charge [43,44]. This technique is characterised by high sensitivity, rapid analysis and relatively low cost (~2 euro to screen up to 40 mutations for one patient) [45]. Sequenom MassARRAY (http://www.sequenom.com) based on MALDI-TOF allows up to 76 thousand genotypes in one day. Only the high costs of the machine explain the low popularity of his technique.

### SNaPshot genotyping

SNaPshot genotyping is a method that incorporates PCR multiplexing. Each DNA sample under analysis is PCR-amplified and subjected to asymmetric PCR reaction where the primer is annealed to the target DNA directly upstream or downstream of the mutation and then extended with DNA polymerase by a single appropriate fluorescent labelled dideoxynucleotide. The product mixture is separated by polyacrylamide gel electrophoresis and analysed by fluorescence detection [46]. Each released product has a specific length that identifies the polymorphic locus and one (in case of homozygotes) or two (in case of heterozygote) of four possible dideoxynucleotides labelled by a specific fluorescence dye that matches the nucleotide at the target site. Visualisation and analysis of the DNA fragments is accomplished by DNA sequencer [47].

According to literature several genetic loci can be simultaneously amplified in a single reaction tube [46-48]. With the capability of high multiplexing, this genotyping method stands out as a robust approach for analysis of known point mutations.

### Strip assay based on primer extension reaction

Dry-reagent strip assay is a novel method, characterised as fast, inexpensive and easy, which enables a visual detection of DNA variants without specialised equipment. Therefore it is a great alternative for small laboratories. Allele discrimination is based on hybridisation of mutant-allele and normal-allele specific products on the nitrocellulose strip [49,50].

Analysis consists of two PCR reactions: DNA amplification and primer extension reaction employing allele-specific primers, dATP, dCTP, dGTP and digoxygenin- and biotin-dUTP (for each allele – ‘normal’ and ‘mutant’) instead of dUTP. Primer extension reaction products are applied to the strip with immobilised anti-digoxigenin and streptavidin, and migrate along by capillary action. Because of the use of gold nanoparticles as reporters, the presence of DNA variants is identified as (one - in case of homozygote -, or two - in case of heterozygote) coloured red spots [49].

The strip assay has proven its diagnostic value as an effective and sensitive method [49,51]. It enables rapid mutation assignment. After PCR amplification visualisation of primer extension reaction products is completed in about 15 minutes. A great advantage is that, as opposed to most genotyping methods, it does not require costly specialised instrument [49]. The only problem could be non-specific binding and misclassifying homo- and heterozygotes but this can be avoided by test optimisation [51]. Nevertheless, strip assay seems to be a noteworthy mutation detection method.

### COLD-PCR

Co-amplification at lower denaturation temperature PCR (COLD-PCR) is a novel modification of the conventional PCR method that selectively amplifies minority alleles from a mixture of wild type and mutant sequences irrespective of the mutation type or position within the sequence [52]. This method is based on the observation that there is a critical denaturation temperature (Tc) for each DNA sequence, which is lower than its melting temperature (Tm). PCR amplification efficiency for a DNA sequence drops abruptly if the denaturation temperature is set below its Tc.

There are two forms of COLD-PCR that have been developed to date: full COLD-PCR and fast COLD-PCR.

Full COLD-PCR includes five stages that are used for each round of amplification:

1. Denaturation – denaturation of the template DNA

2. Intermediate annealing – heteroduplexes formation

3. Melting – melting of heteroduplexes at Tc

4. Primer annealing – annealing of primers to single stranded heteroduplex DNA, homo duplex DNA remain double stranded and is not available for primer annealing

5. Extension – extension of the template DNA by DNA polymerase

In Fast COLD-PCR the denaturation and intermediate annealing stages are skipped.

COLD-PCR is a sensitive platform for the detection of low-abundance mutations and can be used to improve the reliability of a number of different assays that traditionally use conventional PCR, eg. RFLP, sequencing, MALDI-TOF, and real time PCR. Replacing traditional PCR with COLD-PCR for other downstream assays increases the reliability in detecting mutations from mixed samples, including tumors and body fluids.

### Summary

Liquid handling robots have been applied to DNA or RNA isolation, normalisation of sample concentration, PCR preparation, and a variety of other more tedious aspects of mutation detection. The parallel development of software and hardware has enabled complete automatic management of large sample series in genetic testing, including data transfer without any user intervention. Nowadays leading companies offer capillary sequencers capable of analysing simultaneously up to 96 samples amplified by means of cycling sequencing with fluorescent dyes. Improved “chemistry” and capillary gel composition has enabled accurate sequencing of fragments up to 1000 bp in length. Another system Illumina HiSeq 2000-v3 based on massive parallel sequencing by cyclic technology, can generate 600 000 Mb in a single run. Real-time PCR techniques with TaqMan probes have become commonly used in laboratory practice. MLPA technique used in detection of rearrangements in genes associated with hereditary cancers allows the determination of exon copy number. The presence of deletions or duplications of exons or whole genes can be analysed by that method or by the HRMA technique, which also allows simultaneous point mutation detection. Methods like MALDI-TOF or SNaPshot genotyping with the capability of multiplexing enable more cost-effective and less time-consuming testing.

## Competing interests

The authors declare that they have no competing interests.

## Authors’ contributions

GK: contributed to the conception, design, data analyses and manuscript preparation. DD: contributed to literature search and manuscript preparation. PSF: contributed to manuscript preparation. JT: contributed to manuscript preparation. BM: contributed to manuscript preparation. AJ: contributed to manuscript preparation. RJS: contributed to the conception and final manuscript preparation. All authors read and approved the final manuscript.
